# Safety, effectiveness, and usefulness of higher-dose tablets of generic pirfenidone in patients with IPF: a nationwide observational study in South Korea

**DOI:** 10.3389/fphar.2024.1451447

**Published:** 2024-08-09

**Authors:** Jieun Kang, Kwan Ho Lee, Jae Ha Lee, Yi Yeong Jeong, Sun Mi Choi, Ho Cheol Kim, Joo Hun Park, Hyun-Kyung Lee, Suk Joong Yong, Hye Sook Choi, Hak Ryul Kim, Yangjin Jegal, Won-il Choi, Eun Joo Lee, Jin Woo Song

**Affiliations:** ^1^ Division of Pulmonary and Critical Care Medicine, Department of Internal Medicine, Ilsan Paik Hospital, Inje University College of Medicine, Goyang, Republic of Korea; ^2^ Division of Pulmonology and Allergy, Department of Internal Medicine, Yeungnam University Medical Center, Yeungnam University College of Medicine, Daegu, Republic of Korea; ^3^ Division of Pulmonology, Department of Internal Medicine, Haeundae Paik Hospital, Inje University College of Medicine, Busan, Republic of Korea; ^4^ Division of Pulmonology and Allergy, Department of Internal Medicine, Gyeongsang National University Hospital, Gyeongsang National University College of Medicine, Jinju, Republic of Korea; ^5^ Division of Pulmonary and Critical Care Medicine, Department of Internal Medicine, Seoul National University Hospital, Seoul National University College of Medicine, Seoul, Republic of Korea; ^6^ Department of Internal Medicine, Gyeongsang National University Changwon Hospital, Gyeongsang National University College of Medicine, Changwon, Republic of Korea; ^7^ Department of Pulmonary and Critical Care Medicine, Ajou University Hospital, Ajou University School of Medicine, Suwon, Republic of Korea; ^8^ Division of Pulmonary, Allergy, and Critical Care Medicine, Department of Internal Medicine, Busan Paik Hospital, Inje University College of Medicine, Busan, Republic of Korea; ^9^ Department of Internal Medicine, Wonju Severance Christian Hospital, Yonsei University Wonju College of Medicine, Wonju, Republic of Korea; ^10^ Division of Pulmonary, Allergy, and Critical Care Medicine, Department of Internal Medicine, Kyung Hee University Medical Center, Kyung Hee University School of Medicine, Seoul, Republic of Korea; ^11^ Department of Internal Medicine, Institute of Wonkwang Medical Science, Wonkwang University School of Medicine, Iksan, Republic of Korea; ^12^ Division of Pulmonary and Critical Medicine, Department of Internal Medicine, Ulsan University Hospital, University of Ulsan College of Medicine, Ulsan, Republic of Korea; ^13^ Department of Internal Medicine, Myongji Hospital, Hanyang University College of Medicine, Goyang, Republic of Korea; ^14^ Division of Respiratory and Critical Care Medicine, Department of Internal Medicine, Korea University Anam Hospital, Korea University College of Medicine, Seoul, Republic of Korea; ^15^ Department of Pulmonary and Critical Care Medicine, Asan Medical Center, University of Ulsan College of Medicine, Seoul, Republic of Korea

**Keywords:** adherence, adverse events, idiopathic pulmonary fibrosis, lung function decline, pirfenidone

## Abstract

**Background:**

Pirfenidone is an antifibrotic medication approved for idiopathic pulmonary fibrosis (IPF). Fybro^®^, a generic version of pirfenidone developed in South Korea, gained approval and is available in 200 mg and in higher-dose formulations of 400 and 600 mg. This real-world prospective cohort study investigated the safety and effectiveness of Fybro^®^.

**Methods:**

A nationwide observational study was conducted in patients with IPF. Patients were followed up for 6 months, with a subset of patients being followed up for 12 months. Data on lung function and adverse events were collected. Patient adherence to fewer-pill (400 and/or 600 mg tablets) and multiple-pill (200 mg tablets) regimens were compared.

**Results:**

Of the 359 enrolled patients, 352 received pirfenidone (Fybro^®^) at least once and were included in the analysis. The mean age was 69.0 years and 82.4% of patients were male. The median treatment duration was 186.0 days. A total of 253 patients (71.9%) experienced adverse events, with decreased appetite being the most common (16.5%). The adjusted decline rates in lung function were −1.5% and −2.2% predicted per year for forced vital capacity and diffusing capacity, respectively. No significant differences were observed based on the pirfenidone dose. For a daily intake of 1,200 or 1800 mg of pirfenidone, a significantly longer duration of drug administration was observed with the fewer-pill regimen than with multiple-pill regimen.

**Conclusion:**

The safety and effectiveness of Fybro^®^ observed in this real-world cohort study are consistent with previous studies. Using higher-strength tablets to reduce pill burden may improve medication adherence.

## 1 Introduction

Idiopathic pulmonary fibrosis (IPF) is a chronic, progressive, fibrosing interstitial lung disease of an unknown aetiology (Raghu et al., 2018). Prognosis is poor without treatment, with a median survival of 3–5 years after diagnosis (Nathan et al., 2011; Raghu et al., 2014). Antifibrotic medications are widely used to slow the progression of fibrosis (Toellner et al., 2017; Cottin et al., 2018; Margaritopoulos et al., 2018). Pirfenidone, an antifibrotic medication, has demonstrated efficacy in clinical trials by attenuating the decline rate of forced vital capacity (FVC) (Noble et al., 2011; King et al., 2014; Richeldi et al., 2014). Although the precise mechanism of action remains unknown, pirfenidone appears to inhibit TGF-β, TNF-α, and IL-6, and affect the balance of MMPs/TIMPs (Fletcher et al., 2016; Xylourgidis et al., 2019; Ruwanpura et al., 2020). The original form of pirfenidone, Pirespa^®^ (Shionogi & Co), received approval in 2008 in Japan for patients with IPF (Ogura et al., 2015). In South Korea, as in Japan, 1800 mg/day of pirfenidone was approved for the treatment of IPF (Taniguchi et al., 2010). Since then, several generic versions have become available in several countries, including South Korea, China, India, and Mexico. Among the generic versions, Fybro^®^, (Yungjin Pharmaceutical, South Korea) gained approval from the Korean Food and Drug Administration in 2017 based on a dissolution test. Therefore, further assessment of its real-world safety and effectiveness is required.

One notable feature of Fybro^®^ is its availability in the higher-dose formulations of 400 mg and 600 mg whereas Pirespa^®^ is only available in a 200 mg dosage. Achieving the standard daily pirfenidone dose of 1800 mg typically requires patients to be prescribed three tablets of 200 mg, thrice daily. Most patients with IPF are middle-aged or older with comorbidities; therefore, pill burden is a concern (Tzouvelekis et al., 2020; Khor et al., 2022). Therefore, any effort to reduce the pill burden could improve their quality of life. Using 400 mg or 600 mg tablets reduces daily pill count required to achieve a full dose. However, whether reducing pill burden improves medication adherence remains unknown. The objective of this real-world prospective cohort study was to investigate the safety and effectiveness of Fybro^®^ and evaluate whether a treatment regimen utilizing higher-dose tablets to reduce the required pill count can improve medication adherence.

## 2 Materials and methods

### 2.1 Study design and patients

This nationwide observational study was designed to investigate the safety and effectiveness of Fybro^®^. Patients diagnosed with IPF were screened for enrollment at 14 hospitals in South Korea, and those who never received Fybro^®^ were included in the study. The diagnosis of IPF was confirmed through multidisciplinary discussion at each site, based on the American Thoracic Society/European Respiratory Society/Japanese Respiratory Society/Latin American Thoracic Association guidelines (Raghu et al., 2018). Patients were excluded if any of the following conditions were present: 1) hypersensitivity to the main component or additional agent of Fybro^®^, 2) severe or end-stage liver disease, 3) severe or end-stage renal disease, 4) fluvoxamine treatment, and 5) galactose intolerance, Lapp lactase deficiency, or glucose-galactose malabsorption.

The duration of the study was 6 months. For patients who agreed to an extended treatment and follow ups till 12 months, additional data were collected, facilitating a longer-term analysis. The study protocol was approved by the Institutional Review Board of each institution (see ethics statements). All study patients provided written informed consent. This study was registered at Clinical Research Information Service of the Korea National Institute of Health (KCT0008637).

### 2.2 Treatment

Patients were initially prescribed pirfenidone (Fybro^®^) at 200 mg, thrice daily. Depending on their response and tolerance, the dosage was increased by 600 mg every 2 weeks, up to a maximum of 1800 mg per day. In the case of adverse events, the treating physician decided whether to decrease or interrupt the dose, based on event severity. The specific strength (200 mg, 400 mg, or 600 mg) used for administration was determined by the physician.

### 2.3 Data collection

Patients’ baseline clinical characteristics were obtained including age, sex, body mass index (BMI), smoking status, family history of pulmonary fibrosis, IPF diagnosis date, comorbidities, lung function, and minimum oxygen saturation during the 6-minute walk test. IPF severity was categorized using the gender-age-physiology (GAP) index (Ley et al., 2012). The GAP stage is determined based on the total GAP index score: stage I (0–3 points), stage II (4–5 points), and stage III (6–8 points). Lung function parameters, FVC and diffusing capacity of the lung for carbon monoxide (DL_CO_), were measured at baseline and 6 months. The assessment of lung function at 3 months was not mandatory but could be performed based on the treating physician’s judgment. For those who underwent an extended follow up, FVC and DL_CO_ values at 12 months were also collected. Spirometry and DL_CO_ measurements were performed according to the European Respiratory Society/American Thoracic Society guidelines (Macintyre et al., 2005; Miller et al., 2005). Results were presented as percentages of normal predicted values (%pred.).

### 2.4 Study outcomes

Study outcomes were safety and effectiveness of Fybro^®^ in patients with IPF. Adverse events were defined using the preferred terms in the Medical Dictionary for Regulatory Activities version 25.0. The adverse event rates were compared based on background factors, including age, sex, and presence of comorbidities.

To determine the effectiveness of Fybro^®^, decline rates of FVC and DL_CO_ were assessed. Moreover, categorical assessments were conducted for absolute changes in FVC and DL_CO_ at 6 and 12 months. Adherence to fewer-pill regimen (treatment with 400 mg and/or 600 mg tablets) was compared to that of multiple-pill regimen (treatment with 200 mg tablets). Comparisons focused on medication adherence during the periods of administering 1,200 mg/day and 1800 mg/day, respectively. Adherence was evaluated by considering the overall duration of drug usage and frequency of dose modifications. Dose modification was defined as a reduction or temporary interruption in drug dosage, or both, due to intolerance and/or adverse events.

### 2.5 Statistical analysis

Data were presented as mean ± standard deviation or median (interquartile range [IQR]) and numbers (%) for continuous and categorical variables, respectively. The Student’s t-test was used to analyze continuous variables, and the chi-square or Fisher’s exact tests were used to analyze categorical variables. The decline rates of lung function were calculated using a linear mixed model, adjusting for age, sex, BMI, and smoking status. Categorical evaluation of lung function changes was performed in a subgroup of patients who had pulmonary function data available at both baseline and follow ups at 6 or 12 months. Improvement in FVC was defined as an absolute change of +10%pred. or greater; deterioration was defined as an absolute change of −10%pred. or greater, and stability was defined when neither of these criteria were met. Similarly, the absolute changes in DL_CO_ were categorized using a threshold of 15%pred. Logistic regression analyses were performed to analyze the risk factors associated with disease progression, which were defined as a ≥10%pred. absolute decline in FVC and/or ≥15%pred. absolute decline in DL_CO_ at 6 or 12 months. If several variables were observed to be significant in a simple logistic regression (*P*< 0.10) analysis, multiple logistic regression was performed using a backward method. All *P*-values were two-tailed, and a *P*-value of <0.05 was considered statistically significant. All statistical analyses were performed using R version 4.2.2 (R Foundation for Statistical Computing, Vienna, Austria).

## 3 Results

### 3.1 Baseline patient characteristics

**FIGURE 1:**
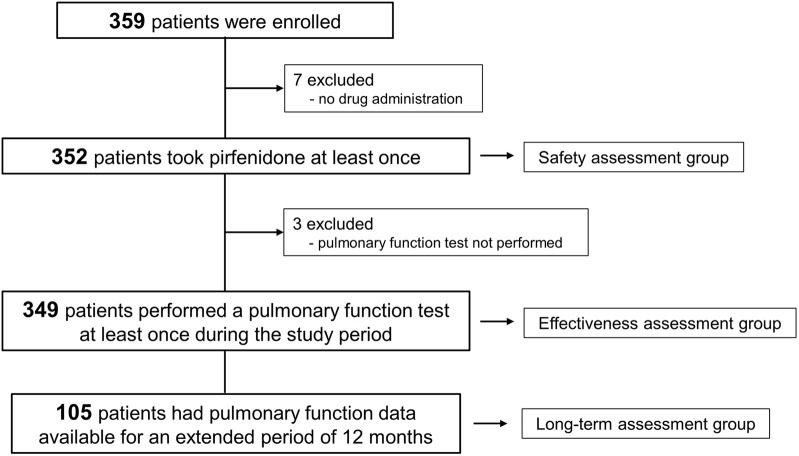
Study flow.

Between November 2018 and September 2021, a total of 359 patients with IPF were enrolled. Among them, 352 patients received pirfenidone at least once and were included in the safety assessment (Figure 1). Pulmonary function data were available for at least one assessment in 349 patients, qualifying them for inclusion in the effectiveness assessment group. Furthermore, pulmonary function data followed up at 12 months were obtained in 105 patients (long-term assessment group).

The baseline characteristics of the 352 patients are summarized in Table 1. The mean age was 69.0 years, and 82.4% were male patients. The median duration of IPF prior to study enrollment was 0.9 (IQR, 0.0–18.0) months. The mean baseline FVC and DL_CO_ were 73.8%pred. and 58.9%pred., respectively. The median duration of the pirfenidone treatment was 186.0 (IQR, 156.0–342.5) days.

**TABLE 1:** Baseline characteristics of study patients.

Category	Characteristics
Number of patients	352
Age, years	69.0 ± 7.5
Male sex	290 (82.4)
Body mass index, kg/m^2^	24.9 ± 3.1
Family history of pulmonary fibrosis	15 (4.3)
Lung biopsy	62 (17.6)
Disease duration, months	0.9 [0.0; 18.0]
Smoking status	
Current smoker	48 (13.6)
Ex-smoker	221 (62.8)
Never smoker	83 (23.6)
Lung function, % of the predicted	
FVC (N = 342)	73.8 ± 16.0
DL_CO_ (N = 336)	58.9 ± 18.9
6MWT minimum SpO2 (%) (N = 326)	90.0 ± 6.3
GAP stage (N = 336)	
1	174 (51.8)
2	143 (42.6)
3	19 (5.7)
Home oxygen therapy	7 (2.0)
Comorbidities	
Hypertension	102 (29.0)
Diabetes	74 (21.0)
Gastroesophageal reflux disease	53 (15.1)
Cardiac disease	42 (11.9)
Chronic obstructive pulmonary disease	33 (9.4)
Dyslipidemia	33 (9.4)
Asthma	26 (7.4)
Chronic liver disease	12 (3.4)
Chronic kidney disease	9 (2.6)
Cerebral infarction	8 (2.3)
Bronchiectasis	7 (2.0)

Data are presented as number (%) or mean ± standard deviation or median [interquartile range].

Abbreviations: FVC, forced vital capacity; DL_CO_, diffusing capacity of the lung for carbon monoxide; 6MWT, 6-min walk test; SpO2, oxygen saturation; GAP, gender-age-physiology.

### 3.2 Adverse events

Among the safety assessment group, a total of 253 patients (71.9%) experienced adverse events (Table 2). The most common adverse event was decreased appetite (16.5%), followed by pruritus (11.9%). No significant differences were observed in the occurrence of adverse events based on sex (72.4%, male; 69.4%, female; *P* = 0.627), age (71.9%, patients aged ≥65 years; 71.9%, patients aged <65 years; *P* > 0.999), or presence of comorbidities (72.3%, with comorbidities; 70.4%, without comorbidities; *P* = 0.731). The frequency of adverse event types did not show significant differences based on sex, age, or comorbidities except for dyspnea which was significantly more frequent in the group without comorbidities than that in the group with comorbidities (18.5% vs. 8.1%, *P* = 0.013) (Supplementary Figure S1 in Supplementary Material).

**TABLE 2:** Adverse events in the safety assessment group.

Adverse events	Number (%)
Number of patients	352
Any adverse events	253 (71.9)
Decreased appetite	58 (16.5)
Pruritus	42 (11.9)
Dyspnea	37 (10.5)
Asthenia	31 (8.8)
Photosensitivity reaction	25 (7.1)
Nausea	23 (6.5)
Dizziness	22 (6.3)
Cough	21 (6.0)
Productive cough	20 (5.7)
Rash	18 (5.1)
Dyspepsia	16 (4.5)
Abnormal liver function test	13 (3.7)
Weight decreased	11 (3.1)
Constipation	10 (2.8)
Idiopathic pulmonary disease	10 (2.8)
Upper abdominal pain	9 (2.6)
Headache	8 (2.3)
Pneumonia	7 (2.0)
Urticaria	7 (2.0)
Diarrhea	6 (1.7)
Vomiting	6 (1.7)
Fatigue	4 (1.1)
Insomnia	5 (1.4)

Data are presented as number (%).

### 3.3 Lung function change

**FIGURE 2:**
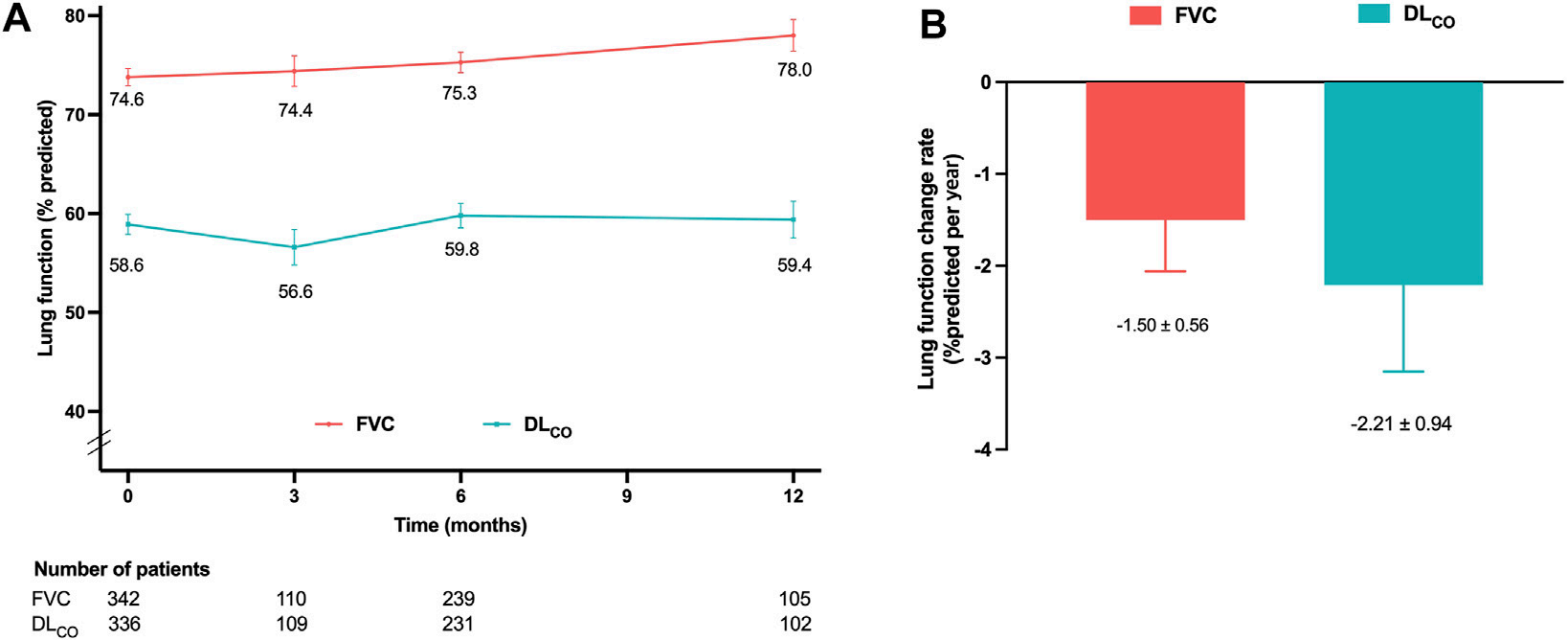
Lung function changes in the effectiveness assessment group **(A)** Mean FVC and DL_CO_ values at 3, 6, and 12 months. **(B)** Decline rates of FVC and DL_CO_ per year adjusted for age, sex, smoking status, and body mass index. Abbreviations: FVC, forced vital capacity; DL_CO_, diffusing capacity of the lung for carbon monoxide.

The decline rates of FVC and DL_CO_ were evaluated in the effectiveness assessment group. The mean values of FVC and DL_CO_ at 3, 6, and 12 months are depicted in Figure 2A. The adjusted decline rates were −1.5%pred. and −2.2%pred. per year for FVC and DL_CO_, respectively (Figure 2B).

**FIGURE 3:**
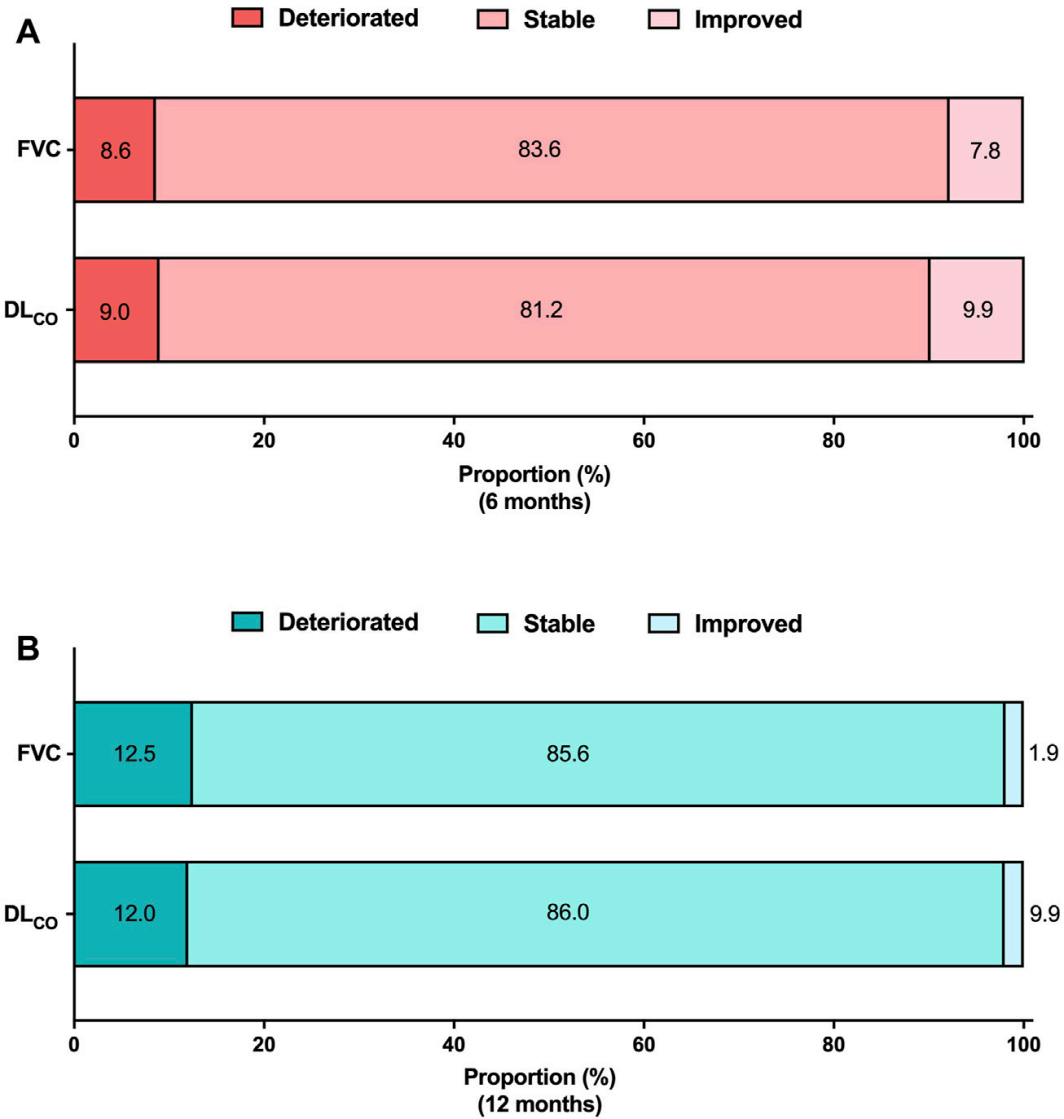
Categorical evaluation of lung function changes Absolute lung function changes **(A)** at 6 months and **(B)** at 12 months. The analysis was conducted on patients who had available pulmonary function data at both the baseline and follow-up periods. FVC 6 months (N = 232), DL_CO_ 6 months (N = 223), FVC 12 months (N = 104), DL_CO_ 12 months (N = 100). Abbreviations: FVC, forced vital capacity; N, number; DL_CO_, diffusing capacity of the lung for carbon monoxide.

Categorical assessment of FVC and DL_CO_ absolute changes at 6 and 12 months is shown in Figure 3. At the 6-month follow-up, 8.6% and 9.0% of patients demonstrated deterioration in FVC and DL_CO_, respectively. Improvements in FVC and DL_CO_ were observed in 7.8% and 9.9% of patients, respectively. The long-term assessment group demonstrated a decline in FVC and DL_CO_ in 12.5% and 12.0% of patients, respectively. Conversely, 1.9% of patients demonstrated an improvement in FVC, and 2.0% demonstrated an improvement in DL_CO_.

### 3.4 Lung function and pirfenidone dose

Patients were categorized into three groups based on the most frequently prescribed dose of pirfenidone during the study period. The low-dose (<1,200 mg/day), medium-dose (1,200 and 1,600 mg/day), and high-dose (1800 mg/day) group consisted of 82, 133, and 134 patients, respectively. Notably, patients in the low-dose group exhibited older age, lower BMI and lung function, and a higher prevalence of advanced GAP stage that those of the patients in the other groups (Supplementary Table S1 in Supplementary Material).

**FIGURE 4:**
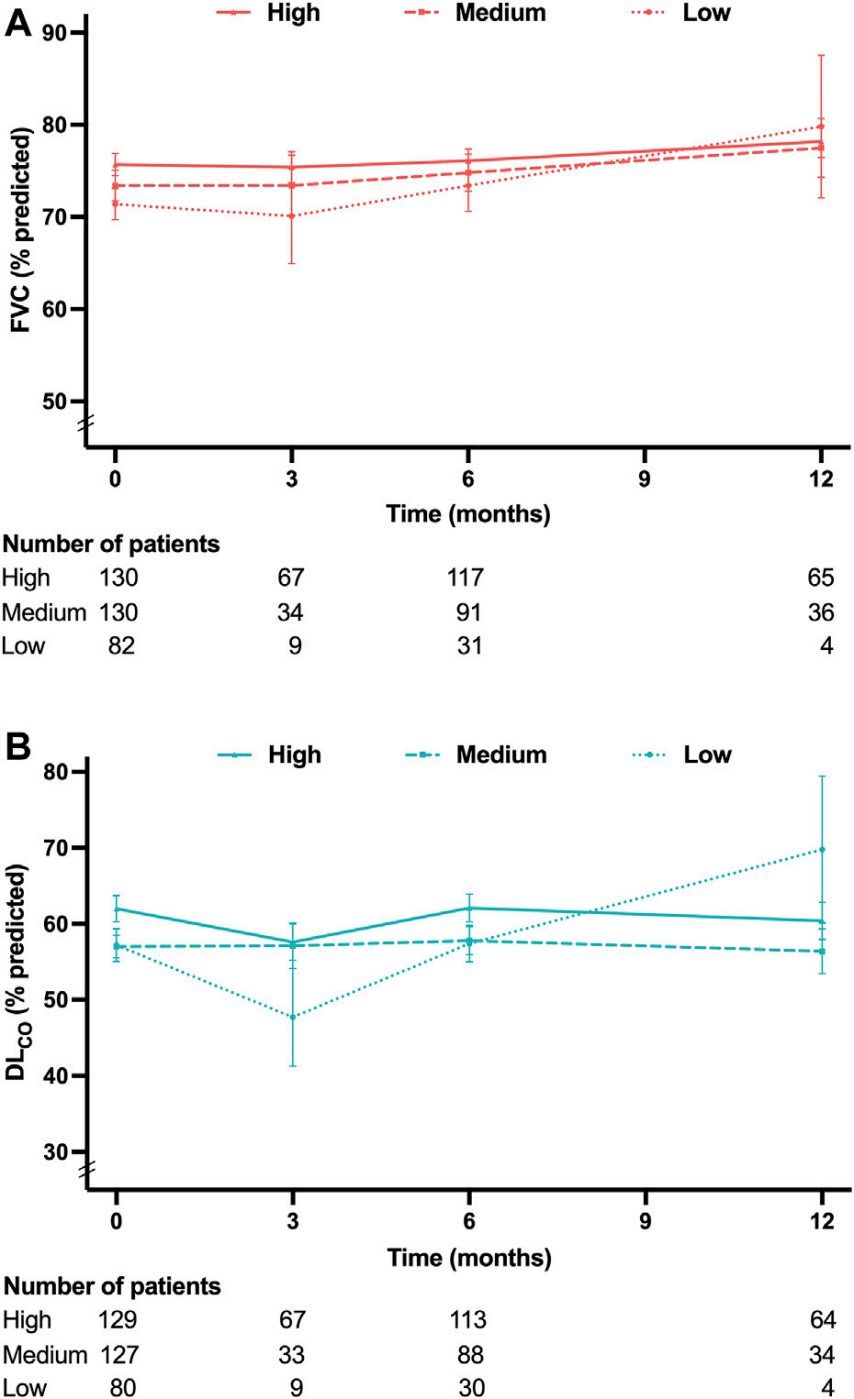
Lung function at each time point according to the pirfenidone dose **(A)** Mean FVC values at 3, 6, and 12 months. **(B)** Mean DL_CO_ values at 3, 6, and 12 months. Pirfenidone dose group was defined as follows: low-dose group, less than 1,200 mg/day; medium-dose group, 1,200 and 1,600 mg/day; and high-dose group, 1800 mg/day. Abbreviation: FVC, forced vital capacity; DL_CO_, diffusing capacity of the lung for carbon monoxide.

**FIGURE 5:**
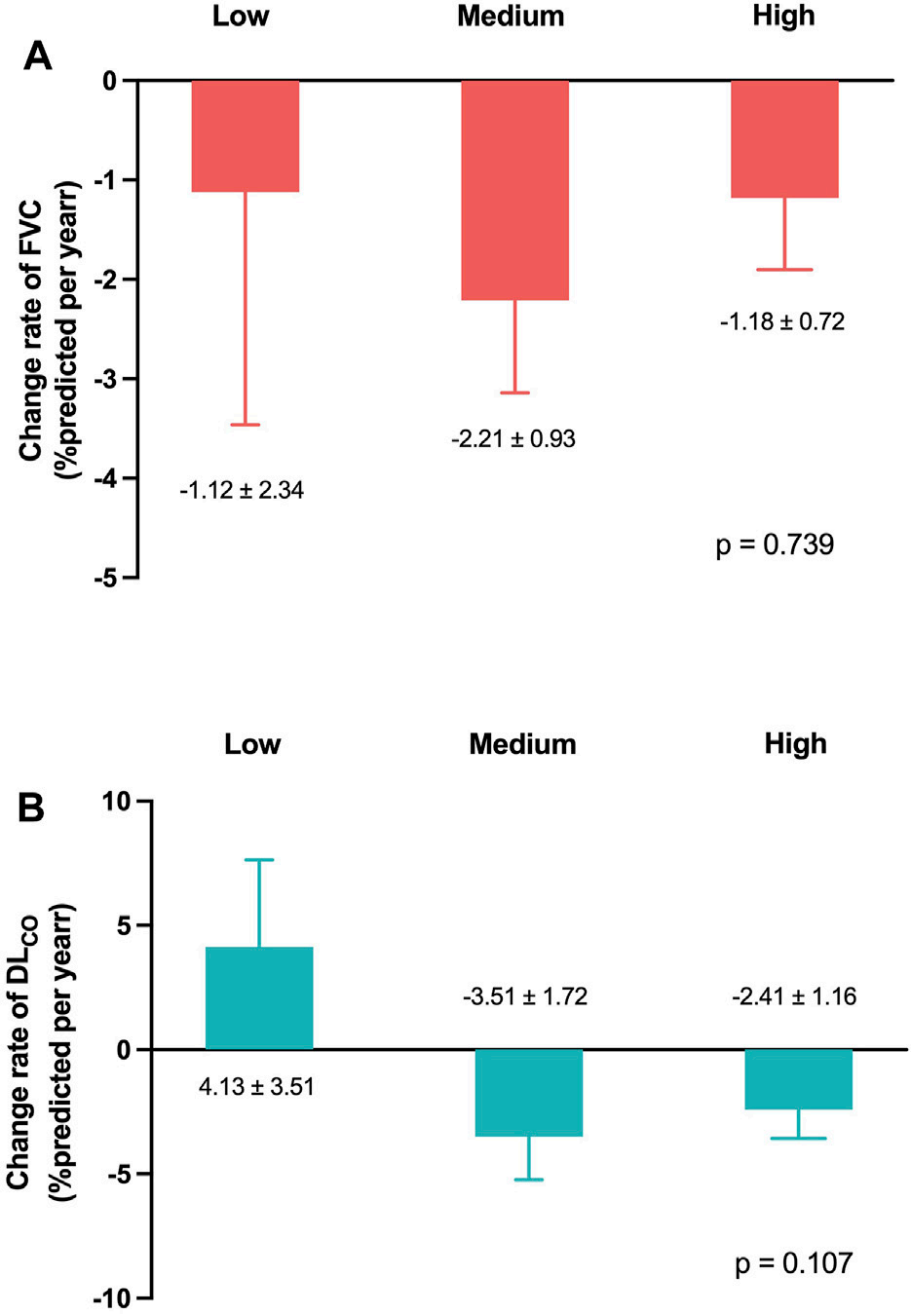
Lung function change rates according to the pirfenidone dose Change rates of **(A)** FVC and **(B)** DL_CO_ per year adjusted for age, sex, smoking status, and body mass index. Pirfenidone dose group was defined as follows: low-dose group, less than 1,200 mg/day; medium-dose group, 1,200 and 1,600 mg/day; and high-dose group, 1,800 mg/day. Abbreviation: FVC, forced vital capacity; DL_CO_, diffusing capacity of the lung for carbon monoxide.

Figures 4A, B present the mean levels of FVC and DL_CO_ at 3, 6, and 12 months according to the pirfenidone dose. The FVC decline rates demonstrated no significant differences among the low-, medium-, and high-dose groups (−1.1%pred., −2.2%pred., and −1.2%pred. per year, respectively; *p* = 0.739) (Figure 5A). Similarly, the DL_CO_ decline rates were not significantly different between the three groups (4.1%pred., −3.5%pred., and −2.4%pred. per year, respectively; *p* = 0.107) (Figure 5B).

### 3.5 Risk factors for disease progression

The risk factors for disease progression at 6 months were analyzed in patients who had pulmonary function data at baseline and 6 months (Table 3). Current smoking demonstrated a tendency towards a higher risk of disease progression at 6 months (odds ratio [OR], 3.27; 95% confidence interval [CI], 0.99–10.74; *p* = 0.051). A higher baseline DL_CO_ value was significantly associated with disease progression (OR 1.02; 95% CI, 1.00–1.04; *p* = 0.043). A longer duration of pirfenidone treatment was significantly associated with a lower risk of disease progression at 6 months (OR, 0.99; 95% CI, 0.99–1.00; *p* = 0.006). According to multivariate analysis, these risk factors remained significant for disease progression at 6 months.

**TABLE 3:** Risk factors for disease progression at 6 Months.

Variables	Unadjusted analysis			Multivariate analysis		
	Odds ratio	95% confidence interval	*P*	Odds ratio	95% confidence interval	*P*
Age	1.03	0.98–1.09	0.223			
Female sex	0.69	0.20–1.90	0.514			
Body mass index	0.99	0.87–1.11	0.823			
Smoking status						
Never smoker	reference	–	–	reference	–	–
Ex–smoker	1.70	0.61–4.76	0.312	1.76	0.61–5.09	0.299
Current smoker	3.27	0.99–10.74	0.051	3.63	1.05–12.57	0.042
FVC	1.00	0.98–1.02	0.934			
DL_CO_	1.02	1.00–1.04	0.043	1.03	1.00–1.05	0.015
PFD duration	0.99	0.99–1.00	0.006	0.99	0.99–1.00	0.001
Pirfenidone dose group^a^						
High dose	reference	–				
Medium dose	0.89	0.40–1.98	0.778			
Low dose	1.36	0.48–3.79	0.563			

Disease progression is defined as ≥10% absolute decline in FVC, and/or ≥15% absolute decline in DL_CO_.

^a^ Pirfenidone dose group is defined as follows: the low-dose group as less than 1,200 mg/day; medium-dose group as 1,200 and 1,600 mg/day; high-dose group as 1800 mg/day.

Abbreviations: FVC, forced vital capacity; DL_CO_, diffusing capacity of the lung for carbon monoxide; PFD, pirfenidone.

The risk factors for disease progression at 12 months were analyzed in patients who had pulmonary function data at baseline and 12 months (Supplementary Table S2 in Supplementary Material). In the unadjusted analysis, only current smoking demonstrated a significantly higher risk of disease progression (OR, 6.67; 95% CI, 1.40–31.72, *P* = 0.017).

### 3.6 Effect of a fewer-pill regimen

A total of 155 patients were identified as having received a dosage of 1800 mg/day of pirfenidone at least once. Among them, 58 patients (37.4%) were administered pirfenidone using the fewer-pill regimen, while 55 patients (35.5%) were administered pirfenidone using the multiple-pill regimen. The remaining 42 patients (27.1%) initially started with an 1800 mg dosage under the multiple-pill regimen; however, subsequently transitioned to the fewer-pill regimen.

To assess the duration of drug administration under each regimen, administration periods were defined as uninterrupted intervals during which patients maintained the same pirfenidone dosage using either the fewer-pill or multiple-pill regimen. Periods without subsequent observations were excluded, as it was not possible to determine dose modifications.

A total of 119 periods were identified for patients treated with the fewer-pill regimen; 103 periods were observed for those using the multiple-pill regimen. The mean duration of drug administration was significantly longer with the fewer-pill regimen than that with the multiple-pill regimen (156.3 vs. 130.0 days; *P* = 0.038). The frequency of dose modification did not show a statistically significant difference between the fewer- (26 cases, 21.8%) and multiple-pill regimens (23 cases, 22.3%). Additionally, no significant difference in the type of dose modification (interruption or reduction) was observed.

A total of 233 patients were identified as having received a 1,200 mg/day dosage at least once. Among them, pirfenidone was administered using the fewer-pill regimen and multiple-pill regimen in 106 patients (45.5%) and 107 patients (45.9%), respectively. The regimen was transitioned from the multiple-pill to the fewer-pill regimen in 17 patients (12.8%), whereas in 3 patients (1.3%) switched from the multiple-pill to fewer-pill regimen and back to multiple-pill regimen. The results were similar among these patients (Table 4).

**TABLE 4:** Medication Adherence According to the pirfenidone regimen.

Regimen	1,800 mg/day			1,200 mg/day		
	Fewer-pill regimen^a^	Multiple-pill regimen^b^	*P*	Fewer-pill regimen^c^	Multiple-pill regimen^d^	*P*
No. of total observed periods	126	111		166	175	
No. of periods excluded from the analysis	7	8		23	9	
Reason for exclusion^e^			0.459			0.397
Follow-up loss	4 (57.1)	7 (87.5)		5 (21.7)	4 (44.4)	
Drop-out	3 (42.9)	1 (12.5)		18 (78.3)	5 (55.6)	
No. of periods included in the analysis	119	103		143	166	
Administration duration, days	156.3 ± 108.9	130.0 ± 73.0	0.038	130.9 ± 109.5	58.7 ± 65.9	<0.001
Dose modification	26 (21.8)	23 (22.3)	>0.999	15 (10.5)	20 (12.0)	0.802
Modification type^e^			0.119			0.612
Interruption	5 (19.2)	0 (0.0)		1 (6.7)	2 (10.0)	
Reduction	20 (76.9)	20 (87.0)		12 (80.0)	12 (60.0)	
Interruption + reduction	1 (3.8)	3 (13.0)		2 (13.3)	6 (30.0)	

Data are presented as number (%) or mean ± standard deviation.

^a^ Fewer-pill regimen for 1800 mg per day includes 600 mg 1T tid (N = 111), 400 mg 1T + 200 mg 1T tid (N = 6), and 400 mg 1.5T tid (N = 2).

^b^ Multiple-pill regimen for 1800 mg per day include 200 mg 3T tid (N = 103).

^c^ Fewer-pill regimen for 1,200 mg per day include 400 mg tid (N = 137) and 600 mg bid (N = 6).

^d^ Multiple-pill regimen for 1,200 mg per day include 200 mg 2T tid (N = 164) and 200 mg 3T bid (N = 2).

^e^ The presented percentage values indicate the proportion of the modification type.

Abbreviations: No, number (referred to in table); N, number (referred to below table); tid, three times per day; T, tablet; bid, twice per day.

## 4 Discussion

In this study, we observed that Fybro^®^ exhibited a similar profile of adverse events and effectiveness as that described in previous reports. The most common adverse event observed was decreased appetite. The decline in the rates of FVC and DL_CO_ were −1.5%pred. and −2.2%pred. per year, respectively. These rates were not significantly different based on the pirfenidone dose. A significantly longer duration of drug administration was observed with the fewer-pill regimen (400 mg and/or 600 mg tablets to achieve daily intake of 1,200 or 1800 mg of pirfenidone) than with multiple-pill regimen.

Pirfenidone’s long-term safety and tolerability has been well-documented. Our study did not identify any new adverse events related to Fybro^®^, and the overall safety profile was similar to that of previous reports. The PASSPORT study (Cottin et al., 2018), which investigated the safety of pirfenidone prospectively over a long-term period, reported that 73.4% of the patients experienced any adverse drug reaction, similar to our findings. However, unlike other studies wherein nausea was reported as the most common adverse event (Lancaster et al., 2016; Noble et al., 2016), our study identified decreased appetite as the most common adverse event. Post-marketing surveillance (PMS) cohort studies conducted in South Korea and Japan also reported decreased appetite to be the most common adverse event (32.4% and 27.9%, respectively) (Ogura et al., 2015; Chung et al., 2020). The differences in the approved doses of pirfenidone in South Korea and Japan, compared with those approved in the United States or Europe, may be related to variations in the safety profile of pirfenidone (Dhooria et al., 2020). Furthermore, variations in adverse event profiles may be influenced by racial differences, indicated by the similar findings in nintedanib studies. In the open-label extension trial of nintedanib (INPULSIS-ON), Asian patients exhibited higher prevalence of decreased appetite than nausea among new initiators (event rate, 14.9 vs. 11.2 per 100 patient exposure-years) (Song J. W. et al., 2020). In addition, an interim report of the nintedanib PMS study conducted in Japan demonstrated that among adverse events leading to drug discontinuation, decreased appetite was more prevalent than nausea (12.7% vs. 5.9%) (Ogura et al., 2023). These findings suggest a lower incidence of nausea and higher incidence of decreased appetite in patients with IPF who are of Eastern Asian origins.

Another notable finding was the relatively lower incidence of gastrointestinal and skin-related adverse events than those reported in previous research (Noble et al., 2011; Ogura et al., 2015; Chung et al., 2020). The CAPACITY trial, a phase III randomized controlled trial evaluating the efficacy of pirfenidone, reported incidence rates of 36%, 32%, and 12% for nausea, rash, and photosensitivity, respectively (Noble et al., 2011). The aforementioned PMS studies conducted in South Korea and Japan also reported higher rates of photosensitivity at 13.7% and 14.4% (Ogura et al., 2015; Chung et al., 2020), respectively, exceeding the 7.1% rate observed in the current study. This discrepancy may be attributed to increased physician awareness of adverse events due to accumulated real-world experience with pirfenidone. Furthermore, enhanced patient education and implementation of preventive measures, such as medication administration with meals and appropriate sunscreen application, may have contributed to the observed difference in adverse events compared with those reported in earlier studies.

The lung function decline rates observed in our study are consistent with findings from previous research (Zurkova et al., 2019; Lee et al., 2021). A retrospective analysis of 383 patients with IPF treated with pirfenidone in the Czech Republic demonstrated annual change rates of 0.2% for FVC and −2.4% for DL_CO_ (Zurkova et al., 2019). The proportions of patients experiencing FVC and DL_CO_ deterioration were also similar with our findings; at 6 months, FVC and DL_CO_ deterioration rates were 5.3% and 6.1%, respectively, while at 12 months, the proportions were 10.7% and 11.3%, respectively (Zurkova et al., 2019). Another recent real-world data study involving 100 patients with IPF reported similar annual change rates of −1.5% for FVC and 0.8% for DL_CO_ (Lee et al., 2021). The similarity of lung function changes to the findings of previous studies provides support for the use of Fybro^®^ in treating IPF. Our analysis of lung function decline based on pirfenidone dose revealed no significant differences between the doses. Previous studies suggested that even with lower doses, the treatment can be effective (Hwang et al., 2022; Kang et al., 2023). In a prospective cohort study involving 143 patients with IPF, no significant differences in lung function changes were observed among the three groups categorized by pirfenidone dose (<1,200 mg, 1,200 mg, and 1800 mg per day) (Kang et al., 2023). Another recent multicenter retrospective cohort study examined the effect of lower-dose pirfenidone on lung function changes before and after treatment (Hwang et al., 2022). The effect of slowing the FVC decline in the lower-dose group was similar to that observed in the standard-dose group.

Our study revealed an association between a longer duration of pirfenidone treatment and lower risk of deterioration in both FVC and DL_CO_. This finding highlighted the importance of implementing strategies that promote long-term pirfenidone use. Previous studies have demonstrated a significant correlation between the number of concomitant medications or polypharmacy at baseline and antifibrotic medication intolerance within the first 6 months in patients with IPF (Khor et al., 2022). Although the study did not specifically consider the number of pirfenidone tablets, this finding implies a potential relationship between higher pill burden and pirfenidone intolerance. Therefore, improving the convenience of administration by reducing tablet counts may increase medication adherence. Our study observed a significant association between fewer-pill regimen for the same dose (1,200 or 1800 mg per day) and prolonged treatment duration than that with a multiple-pill regimen. However, our study was not explicitly designed to address this issue; therefore, establishing a direct causal relationship between fewer pirfenidone tablets and patient adherence is not possible. As certain patients switched from a multiple-to a fewer-pill regimen, a bias towards prolonged administration in the latter regimen could exist due to their inclusion of well-tolerated patients receiving 1800 mg dosage under the multiple-pill regimen. Further research is warranted to examine the potential advantages of a pirfenidone regimen with fewer pills.

Pirfenidone has also been investigated for its efficacy in progressive pulmonary fibrosis (PPF) (Tzilas et al., 2020; Behr et al., 2021). A double-blind, randomized controlled phase 2b trial investigating pirfenidone in progressive fibrotic interstitial lung disease revealed that the pirfenidone group exhibited a significantly smaller decline in FVC %pred. from baseline to week 48 compared to the placebo group (Behr et al., 2021). The result should be interpreted with caution due to the premature termination of the study as a result of slow recruitment and further research is required (Raghu et al., 2022). Nevertheless, the clinical similarities between PPF and IPF suggest that pirfenidone may also be beneficial for PPF. The indications for Fybro might be extended beyond IPF to encompass PPF, leading to broader application of the drug. The effectiveness and safety of Fybro demonstrated in our study could provide valuable guidance for clinicians in the prescribing of this medication.

Our study had some limitations. First, the study duration was 6 months, and only a subset of patients was followed up till 12 months. Categorical evaluation of lung function changes and logistic regression analysis to identify risk factors for lung function deterioration at 12 months may have been underpowered. Although we utilized linear mixed models to calculate lung function change rates, further studies including a larger sample are warranted. Second, our study did not evaluate important clinical outcomes, such as acute exacerbation and mortality. Despite the expectation of Fybro^®^ exhibiting similar positive effects on these outcomes, considering its demonstrated effectiveness in preventing lung function decline observed in our study, additional research with a specific focus on these outcomes is required. Third, the number of patients included in the low-dose group was relatively small than that in the other groups, which may have influenced the calculated lung function decline rate in this group. However, our results indicated no significant difference in the effects of lower doses of pirfenidone, aligning with those of previous research (Song M. J. et al., 2020; Hwang et al., 2022). Finally, our study was conducted in patients treated with Fybro^®^, a medication developed in South Korea. The results may not be readily generalized to other generic versions of pirfenidone and further studies are needed.

In conclusion, the safety and effectiveness of Fybro^®^ investigated in a real-world prospective cohort study were similar to those reported in previous studies. Using higher-dose tablets to reduce the required pill count for dose achievement may enhance medication adherence.

## Data Availability

The raw data supporting the conclusions of this article will be made available by the authors, without undue reservation.

## Abbreviations

BMI, body mass index; CI, confidence interval; DLCO, diffusing capacity of the lung for carbon monoxide; FVC, forced vital capacity; GAP gender-age-physiology; IPF, idiopathic pulmonary fibrosis; IQR, interquartile range; OR, odds ratio, PMS, post-marketing surveillance.
