# Supplementary material for: Safety, effectiveness, and usefulness of higher-dose tablets of generic pirfenidone in patients with IPF: a nationwide observational study in South Korea
Source: Front Pharmacol. 2024 Aug 9;15:1451447. doi: 10.3389/fphar.2024.1451447 (PMC11341391; doi:10.3389/fphar.2024.1451447)
Supplement: Supplementary file 1 [file DataSheet1.docx]

**Additional File 1**

**Safety, effectiveness, and usefulness of higher-dose tablets of generic pirfenidone in patients with IPF: a nationwide observational study in South Korea**

**Figure S1. Frequency of adverse events based on sex, age group, and presence of comorbidities**

**
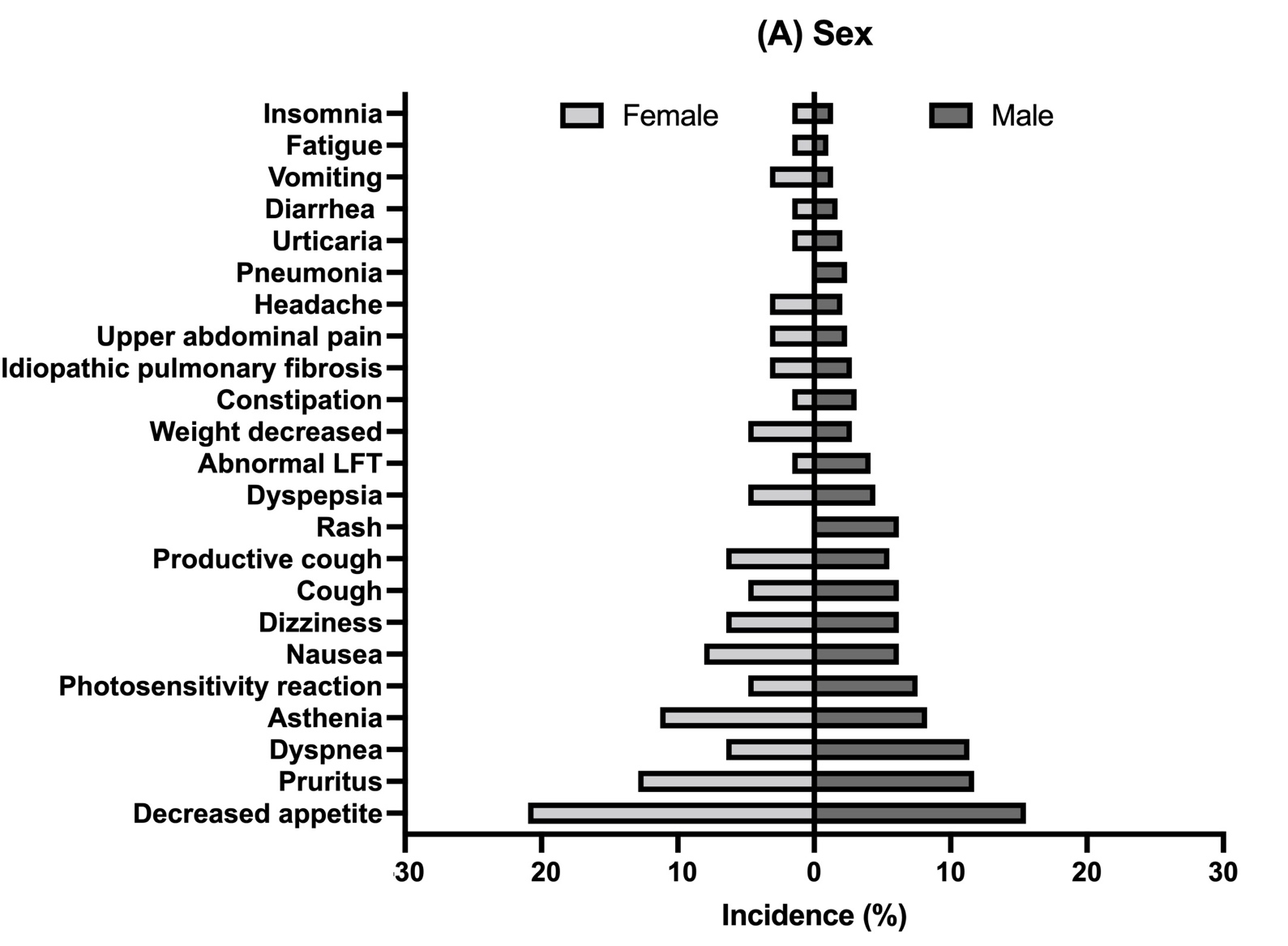
**

**
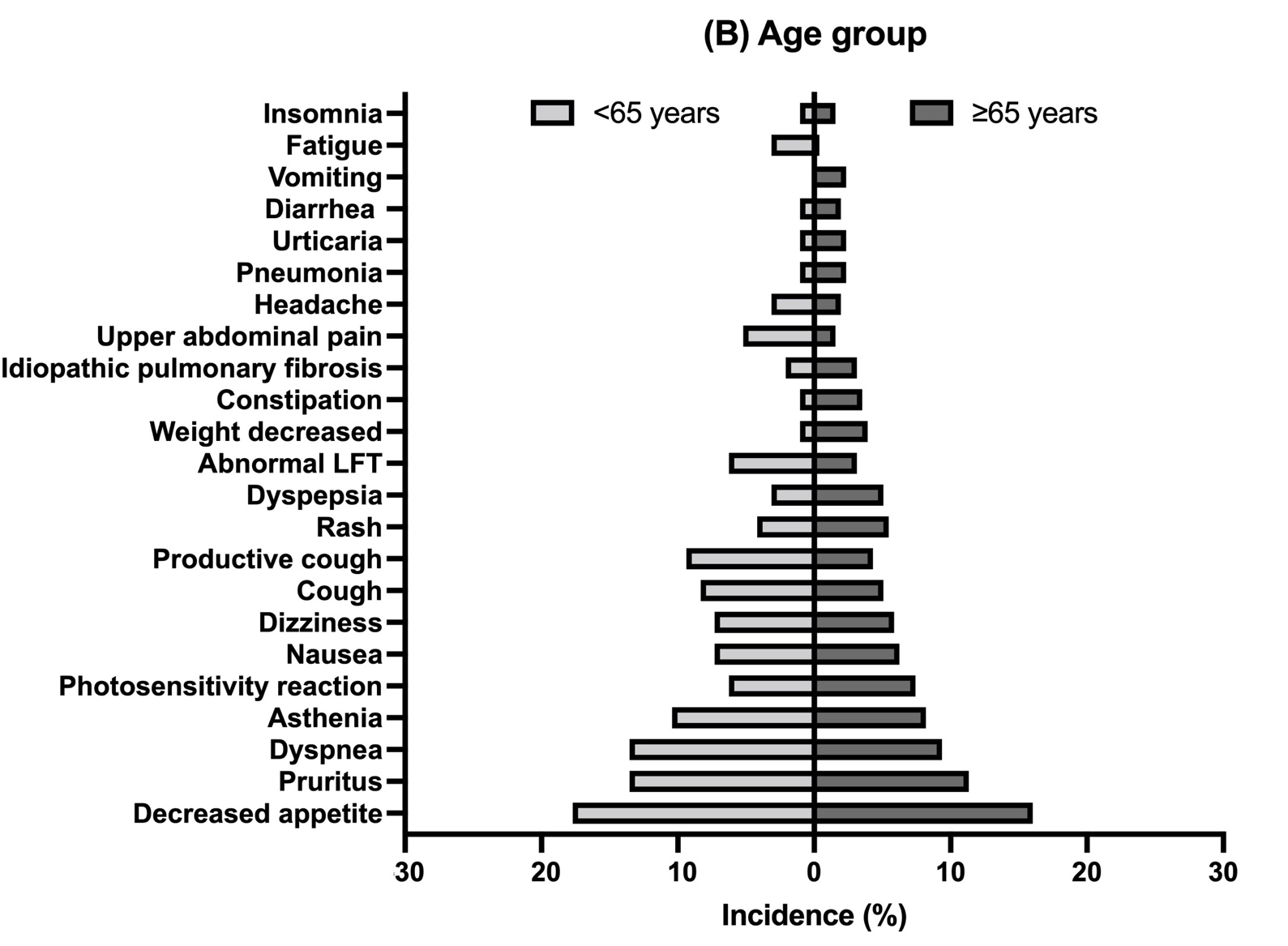
**

**
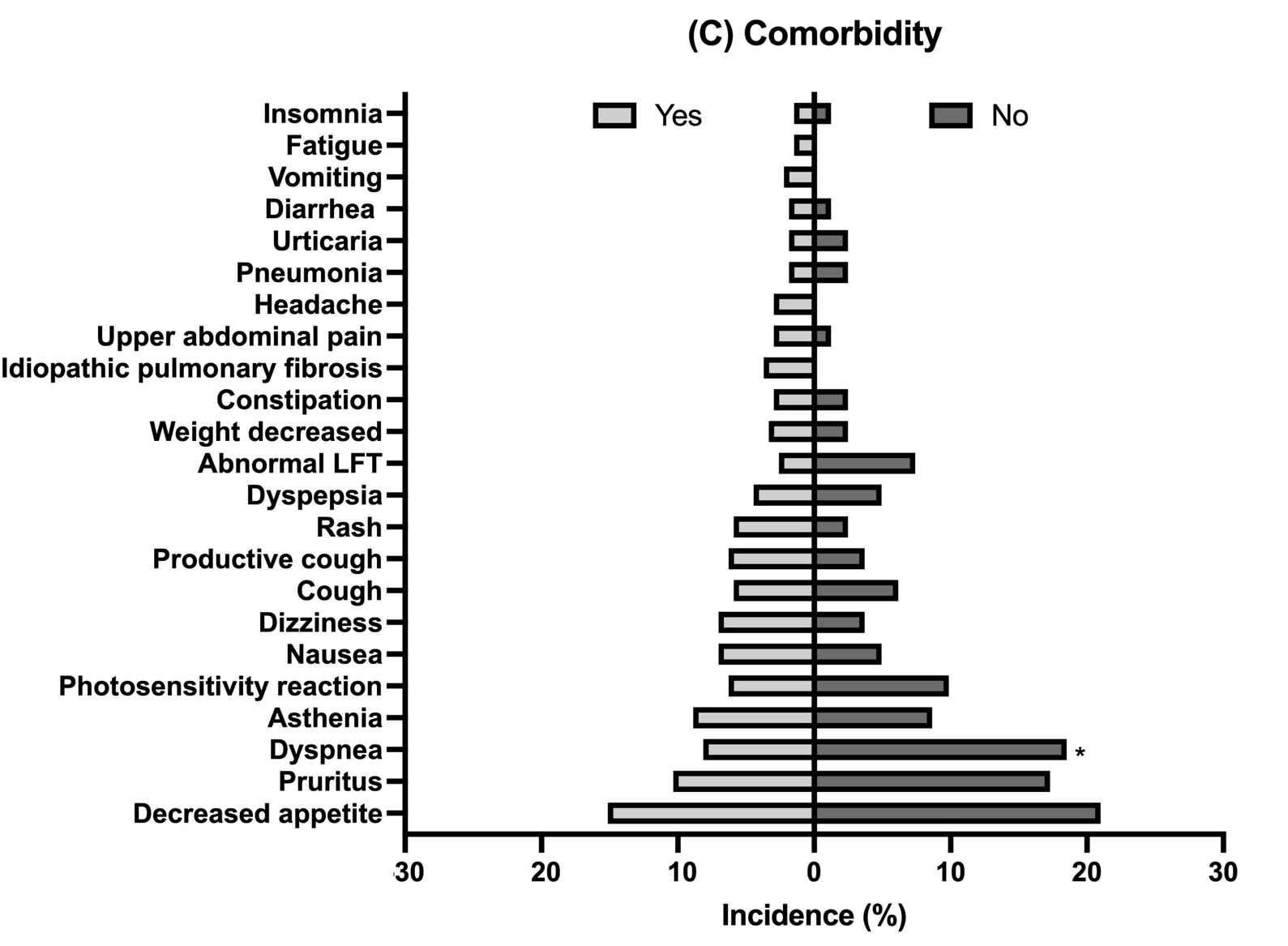
**

The frequency of adverse event types did not show significant differences based on (A) sex, (B) age, or (C) the presence of comorbidities except for dyspnea which was significantly more frequent in the group without comorbidities than in the group with comorbidities (18.5% vs. 8.1%, *P*=0.013). Abbreviation: LFT, liver function tests

* indicates *P*<0.05.

**Table S1 Baseline Characteristics of Study Patients According to the Pirfenidone Dose**

| Variable | Low dose | Medium dose | High dose | *P* |
| --- | --- | --- | --- | --- |
| Number of patients | 82 | 133 | 134 |  |
| Age, years | 71.1 ± 6.9 | 69.2 ± 7.7 | 67.5 ± 7.3 | 0.002 |
| Male sex | 64 (78.0) | 108 (81.2) | 116 (86.6) | 0.244 |
| Body mass index, kg/m^2^ | 24.1 ± 3.0 | 24.7 ± 3.0 | 25.4 ± 3.3 | 0.011 |
| Disease duration, months | 2.0 [0.0;21.2] | 1.0 [0.2;19.8] | 0.6 [0.0;12.9] | 0.363 |
| Smoking status |  |  |  | 0.700 |
| Current smoker | 10 (12.2) | 15 (11.3) | 23 (17.2) |  |
| Ex-smoker | 52 (63.4) | 85 (63.9) | 81 (60.4) |  |
| Never smoker | 20 (24.4) | 33 (24.8) | 30 (22.4) |  |
| Lung function, % of the predicted |  |  |  |  |
| FVC | 71.4 ± 14.7 | 73.4 ± 18.4 | 75.7 ± 14.0 | 0.143 |
| DL_CO_ | 57.2 ± 19.8 | 57.0 ± 17.2 | 62.0 ± 19.6 | 0.068 |
| 6MWT minimum SpO_2_ (%) | 88.7 ± 7.1 | 90.2 ± 6.0 | 90.7 ± 6.1 | 0.106 |
| GAP stage |  |  |  | 0.077 |
| 1 | 32 (40.0) | 66 (52.0) | 76 (58.9) |  |
| 2 | 41 (51.2) | 53 (41.7) | 49 (38.0) |  |
| 3 | 7 (8.8) | 8 (6.3) | 4 (3.1) |  |
| Total treatment duration, days | 130.5 [31.0;176.0] | 184.0 [168.0;336.0] | 319.5 [183.0;377.0] | <0.001 |
| Average dose, mg per day | 667.1 ± 141.0 | 1178.0 ± 107.5 | 1657.9 ± 126.2 | <0.001 |

Data are presented as number or mean ± standard deviation.

Abbreviation: FVC, forced vital capacity; DL_CO_, diffusing capacity of the lung for carbon monoxide; 6MWT, 6-minute walk test; SpO_2_, oxygen saturation; GAP, gender-age-physiology.

**Table S2 Risk Factors for Disease Progression at 12 Months**

|  | Unadjusted analysis | | |
| --- | --- | --- | --- |
| Variables | Odds ratio | 95% confidence interval | *P* |
| Age | 1.06 | 0.99–1.14 | 0.136 |
| Female sex | 0.88 | 0.19–3.09 | 0.856 |
| Body mass index | 0.89 | 0.73–1.07 | 0.249 |
| Smoking |  |  |  |
| Never smoker | reference | – | – |
| Ex–smoker | 1.07 | 0.26–4.36 | 0.923 |
| Current smoker | 6.67 | 1.40–31.72 | 0.017 |
| FVC | 1.02 | 1.00–1.06 | 0.113 |
| DL_CO_ | 1.00 | 0.97–1.03 | 0.964 |
| PFD duration | 1.01 | 0.99–1.02 | 0.491 |
| Average dose of PFD | 1.00 | 1.00–1.00 | 0.873 |
| Frequently used dose PFD | 1.00 | 1.00–1.00 | 0.789 |
| Nonstandard-dose PFD | 1.40 | 0.51–3.76 | 0.505 |
| Pirfenidone dose |  |  |  |
| High dose | reference | – | – |
| Medium dose | 1.61 | 0.59–4.35 | 0.351 |
| Low dose | 0.00 | 0.00–infinite | 0.990 |

Abbreviation: FVC, forced vital capacity; DL_CO_, diffusing capacity of the lung for carbon monoxide; PFD, pirfenidone.
